# Facilitators and barriers in diagnosing rheumatoid arthritis as described by general practitioners: a Danish study based on focus group interviews

**DOI:** 10.1080/02813432.2021.1913925

**Published:** 2021-04-27

**Authors:** Anne Sofie Baymler Lundberg, Bente Appel Esbensen, Martin Bach Jensen, Ellen Margrethe Hauge, Annette de Thurah

**Affiliations:** a Department of Clinical Pharmacology, Aarhus University Hospital, Aarhus, Denmark; b Research Unit for General Practice, Department of Public Health, Aarhus University,Aarhus, Denmark; c Copenhagen Center for Arthritis Research (COPECARE), Center for Rheumatology and Spine Diseases, Center of Head and Orthopedics, Rigshospitalet, Denmark; d Department of Clinical Medicine, Faculty of Health and Medical Sciences, University of Copenhagen, Denmark; e Center for General Practice at Aalborg University, Aalborg, Denmark; f Department of Rheumatology, Aarhus University Hospital, Aarhus, Denmark; g Department of Clinical Medicine, Aarhus University, Aarhus, Denmark

**Keywords:** Rheumatoid arthritis, primary health care, focus group interviews, early diagnosis, patient delay

## Abstract

**Objective:**

To explore the perspectives of general practitioners (GPs) on facilitators and barriers in diagnosing rheumatoid arthritis (RA).

**Design:**

Qualitative study based on focus group interviews, and using latent thematic analysis.

**Setting:**

General practices from Central Region Denmark.

**Subjects:**

Eleven GPs participated in three different focus groups. Forty percent were female, the mean age was 53 years (range 37–64), and the mean since medical licensing was 16 years (range 5–23). Sixty percent of the GPs worked in an area served by a university hospital, and 40% were served by a regional hospital.

**Main outcome measure:**

Themes describing experiences and reflections about facilitators and barriers in diagnosing Rheumatoid Arthritis.

**Results:**

Four themes emerged: (A) If the patient is not a textbook example, (B) The importance of maintaining the gatekeeper function, (C) Difficulties in referral of patients to the rheumatologist, and (D) Laboratory tests—can they be trusted? Barriers were identified in all themes, but facilitators only in A, C, and D. The overarching theme was *Like finding a needle in a haystack*.

**Conclusion:**

The GPs found several barriers for diagnosing RA (symptom awareness, GP’s gatekeeper function, suboptimal collaboration with rheumatologists and limitations in laboratory tests). They identified education, more specific tests and better access to rheumatologists as possible facilitators for diagnosing RA. To facilitate earlier referral of suspected RA in general practice and strengthen mutual information and collaboration, future research should focus on these facilitators and barriers.

**Keypoints**
- Early diagnosis is essential for the prognosis of RA, and the diagnostic process begins in general practice.
- Suggested facilitators: training courses in interpretation of laboratory tests and the clinical manifestation of RA, and videos on joint examinations.
- Suggested barriers: compliance with the gatekeeper function, suboptimal collaboration with rheumatologists, limitations of laboratory tests, and diversity of clinical manifestations.

## Introduction

Rheumatoid arthritis (RA) is a chronic inflammatory joint disease with a prevalence of approximately 5 per 1000 adults. Previously, RA often had devastating consequences with permanent joint damage, considerable disability, and systemic manifestations. Although the new drugs and treatment escalations (the so-called treat to target strategy [1]) have markedly improved the long-term prognosis [1,2], early treatment in the first 3 months from RA symptom onset is essential to increase the likelihood of remission and to prevent permanent joint damage [2].

Unfortunately, treatment often starts later than 3 months after symptom onset [3–5]. A 2017 review found the median lag time from symptom onset to therapy was 11 months [6]. A Danish study based on data from the clinical database, DANBIO (Danish Registry for Biologic Therapies in Rheumatology) found that the mean delay in 2009–11 were 6 months. The results were however, biased by missing information in 28% of the cases [7]. A study from 2019 identified diagnostic delay at all levels in the pathway toward assessment by a rheumatologist. Only 20% of patients were seen within the first three months after symptom onset, with general practice accounting for 6.9 weeks out of a total 27.2 weeks of diagnostic delay [4].

In some healthcare systems, e.g. Denmark and the UK, general practitioners (GPs) play a key role in diagnosing RA, as they typically serve as gatekeepers for specialist care, such as rheumatologists [8,9]. In general practice, identifying patients who need to be referred to rheumatologists for suspected RA is difficult for several reasons. First, RA is an uncommon disease: approximately 1300 new RA cases are identified annually in Denmark, and, with around 3400 GPs, a Danish GP encounters on average only about one new patient with RA every 3 years. Second, RA is a clinical diagnosis; there are no blood tests or imaging that can rule out RA with certainty [10,11]. Third, the onset of RA is often insidious, and early-stage joint swelling can be asymmetric or monoarticular [2].

In a qualitative study from Belgium, GPs’ beliefs and experiences regarding early referral of patients for RA suspicion were explored. They described various barriers for referral, including low confidence in detection, limited access to specialists, and poor professional collaboration between sectors [12]. This complexity in causes of delay, including limited knowledge of RA among GPs, was also reported in a study from the United Kingdom [13] and a review from 2017 [6].

To the best of our knowledge, no study has addressed possible facilitators in diagnosing RA. A Danish study examined patient perspectives on a possible diagnostic delay in RA [14] but did not focus on GP perspectives. The development of interventions to support GPs in earlier detection of RA needs to be based on solid knowledge about both facilitators and barriers seen from the GPs’ perspective.

### Objective

To explore GPs’ perspectives on facilitators and barriers in diagnosing RA.

## Methods

### Design

This was a qualitative study based on three semi structured focus group interviews with 11 GPs from the Central Denmark Region.

### Setting

Denmark has 5.4 million inhabitants, and all residents have free and direct access to GPs [15]. The healthcare system in Denmark is public and financed through taxes, and all patients, except those who choose otherwise, are listed with a GP for primary healthcare. The GPs have a gatekeeper function, where patients are referred to secondary care as decided by the GPs. The exceptions are emergency service, ophthalmologists, and otorhinolaryngologists, whom the patients can consult directly. Treatment is free of charge for patients; and GPs are paid by a combination of capitation and free-for-service reimbursement [15].

### Participants

GPs working in the Central Denmark Region (*n* = 812) were eligible to participate in the focus group interviews [16]. To recruit participants, electronic information was sent as a newsletter through the webpage *Praksis.dk*, an information portal targeted at GPs in the Central Denmark Region. GPs who were willing to participate contacted the study leader (AdT) via e-mail.

The GPs signed an informed consent and were reimbursed for their participation.

### Procedure

First, the research team identified possible facilitators and barriers for GPs in the RA diagnostic process from a systematic literature review, and an interview guide was developed. The moderator (AdT, health services researcher, MPH, PhD) is senior researcher at the Department of Rheumatology, Aarhus University Hospital, and the observer (ASL, MD) is a GP in training (one and a half year of experience at three different general practices). AdT has many years’ clinical experience within rheumatology as well as research experience in RA and has attended a focus group session among GPs as an observer at the Center for General Practice at Aalborg University. The conduction of the interview guide as well as the coding and interpretation of results was supervised by BAE, who has an extended expertise within qualitative research.

Only the names and titles of the moderator and observer were presented to the participants. The participants had not received any information about the project, and the diagnostic process of RA was the focus of the interview. Neither the moderator nor the observer knew the participants professionally or privately. At the start, the participants were offered the opportunity to narrate their experience, through the opening invitation [17]: ‘Please recall your last patient diagnosed with RA and tell us about that’. This was done in order to set the scene for the interview, and stimulate the participants focus on the topic [18]. Interviews were conducted until saturation was achieved. Table 1 shows the themes and questions and the planned structure of the focus group interviews.

**Table 1.** Interview guide.

*Pre-interview: brief information*
1: Recall a patient
*Please recall your last patient diagnosed with RA and speak about that.*
2: Incidence locally
*How often do you see a patient with newly diagnosed RA in your general practice?*
3: GPs’ experience
*What is your experience with diagnosing RA?*
4: Availability of resources
*What are your experiences with available paraclinical testing?*
5: Referral to the secondary sector
*When do you choose to refer to a rheumatologist?*
*What are your experiences with referral to rheumatologists?*
*What are the drivers and barriers in early referral to rheumatologists?*
6: Knowledge of treat to target
*What are your opinions on the treat-to-target strategy?*
7: GPs’ role in diagnosing RA
*How can a GP play a part in the early diagnosis of RA?*
*What are your experiences of collaboration with secondary care?*
*What are the facilitators and barriers to follow the treat-to-target strategy?*
8: Closure
*Is there anything missing according to the subject?*

Each focus group interview lasted a maximum of one hour and took place in a meeting room at the Department of Rheumatology, Aarhus University Hospital. All interviews were recorded digitally.

After each interview, the moderator and observer shared their initial impressions. The moderator transcribed the interviews verbatim, and the moderator and the observer reviewed all the transcripts. The moderator uploaded all the transcripts to NVivo v.12 for analysis. Latent thematic analysis was applied as described by Braun and Clarke [19] by continuously asking in which way did the GPs experience facilitators and barriers in diagnosing patients with RA in general practice. This approach was inductive, which entails the identified themes as strongly connected to the data. After generating initial codes, we reviewed four themes in relation to the coded extracts and generated a thematic map. The themes were subsequently defined and labeled by identifying each theme’s essence [19]. Lastly, we selected vivid examples from the transcripts. An independent researcher, who is experienced in qualitative medical research (BAE), reviewed the transcripts and commented on the themes that emerged after the coding process. Any discrepancies in the analytical process were resolved through discussion until consensus among all authors was obtained.

The analysis was conducted in line with the Consolidated Criteria for Reporting Qualitative Research (COREQ) [20].

### Ethics

According to Danish law, approval by the regional committee on health research ethics was not required, as no biomedical intervention was performed. The study was approved by the regional data protection agency (ref no: 1-16-02-199-20). The study complies with the Declaration of Helsinki’s ethical principles.

The discussion was only about general views and perspectives on the topic, i.e. the GPs did not reveal any information that could be connected to real patients.

All participants gave written consent. All personal data was anonymized when transcribed. The GPs were not offered the possibility of commenting on the results.

## Results

Three focus group interviews were held with respectively four, four, and three GPs in each group during spring and autumn 2019. The mean age was 53 years (range 37–64), 40% of the participants were female, and the mean year since specialization as a GP was 16 years (range 5–23). Most participants were not acquainted, besides two who were connected professionally, as they came from the same practice. Regarding general practice location, 60% of the GPs worked in a practice located within a university hospital’s referral area and the rest within a regional hospital’s referral area. Ten worked in a general practice with three or more doctors, and only one participant worked in a solo practice. Data saturation was reached after three focus group interviews, meaning that no additional data were found for new themes [21].

### Themes

The thematic analysis resulted in an overarching theme and four themes (A–D), which are listed in Table 2 together with selected quotes from the GPs as examples of each theme.

**Table 2.** The four themes A–D with numbered quotes illustrating each theme.

Barriers	Facilitators
*Theme A If the patient is not a textbook example*	
A1 ‘It is rare when I see someone where I think—this is truly RA.’	A8 ‘To examine a finger joint—when is there arthritis?’
A2 ‘If it was only like in the textbooks THEN it would be easy.’	A9 ‘But I think, it could be a topic—as you say—many new things have happened, and the approach has changed [on diagnosing RA]; therefore it could be an area of training [for specialized doctors].’
A3 ‘The debut is so varied. It is not necessarily a textbook example. It is rare that it is like that.’	A10 ‘A 30-second video.. like when you find a younger doctor [at the department of clinical rheumatology] to illustrate.’
A4 ‘I cannot remember looking at a patient’s fingers and thinking oh my God this is classic RA.’	A11 ‘I could dream of that at your webpage for health professionals; maybe sometimes there could be some pictures, a little video.’
A5 ‘Sometimes they [patients] say it is swollen, and you cannot see it, but they are certain. There are some problems, I think. Because if it was bigger, red, asymmetrical, then you have no doubts.’	
A6 ’It is difficult when there is no arthritis.’	
A7 ‘When we miss something, it is the patients, who come too early to suspect RA.’	
*Theme B The importance of maintaining the gatekeeper function*	
B1 ‘We find cancer earlier now, but it has a cost. We use more resources on examining people. How many resources does it take to find the sero-negative [RA patients]?’	
B2 ‘I have become more proficient with age. And there lies a—you should not overuse the healthcare system.’	
B3 ‘I know I am good at gynecology, and I know a lot about lung diseases, but all of [a] sudden, you [the rheumatologists] need to take this, because I do not know much about it, and I am aware of that, so I will refer earlier [than other GPs].’	
*Theme C Difficulties in referral of patients to rheumatologists*	
C3 ‘I get a call from the rheumatologist. Is the list really that long? Or what is the problem since I am interrupted in [the] middle of a patient [consultation], to answer why I have referred a patient? I think that is not acting as a good colleague.’	C1 ‘I think if there is arthritis the patients are seen within a week.’
C4 ‘If the blood tests are positive, then they accept them. They do! But it is the gray areas, that are the difficult ones.’	C2 ‘But I think in xx [city name], they must be praised for saying: ‘Bring them, and we can quickly handle and finish it.’
C6 ’We can often get a phone call from the specialist doctor the next day [after referral], advising us to try this and that, so because of that we often chose the private [practice] rheumatologists more.’	C5 ‘The access [to rheumatologists] is easy if the biomarkers are clear.’
C7 ‘The private [practice] rheumatologists, are more when I think, No it is probably nothing, but there is a big wish [from the patient to be referred] or when it has been going on for a long time.’	
C8 ‘When they [patients] have been to a rheumatologist before, I really think that it is a barrier, if they are declared ‘free’ [from a rheumatic disease].’	
*Theme D Laboratory tests—can they be trusted?*	
D5 ‘There is a big difference in how to interpret the blood samples—what do we know about blood samples? I did not know anything about the sensitivity on that thing [Anti–citrullinated protein antibody (ACPA)] and we can miss a lot of patients, so we need information about that. I don’t mind admitting that during the diagnosing process, I have to read about the blood tests and the diagnosis.’	D1 ‘The younger colleagues take a big amount of ‘rheuma-tests’ and everything, where I know for sure that they do not have RA.“
	D2 ‘I can tell, what I am trying to tell my intern doctors, that they [the patients] must have a swollen joint, then we can take a CRP [C-reactive protein] and if it is normal, [and] so on and so on, then they do not have RA, then they definitely need to look for something else.’
	D3 ‘It is much easier after the ACPA occurred.’
	D4 ‘The ACPA should be more sensitive. There is also the rheuma factor-negative RA which complicates things […] the ACPA should be more sensitive—that is my impression.’

The overarching theme was *Like finding a needle in a haystack*, covering the GPs’ difficulties in detecting RA, because patients’ symptoms often resembled those of more common and less serious conditions, such as benign musculoskeletal complaints, i.e. injuries after excessive straining.

#### Theme (A) if the patient is not a textbook example

The diagnosis was difficult to suspect due to different manifestations, which are not always as described in textbooks (Quotes A1, A2, A3, and A4). The participants especially found it difficult to diagnose if they thought there were no clear swollen joints (Quotes A5, A6, and A7).

Some GPs also discussed possible drivers, which were that the diagnosis of RA could be a topic for training courses for specialized GPs (Quotes A8 and A9). Videos with joint examination were other suggested facilitators for helping diagnose arthritis (Quotes A10 and A11).

#### Theme (B) the importance of maintaining the gatekeeper function

In Denmark, GPs have an important task to select patients who need treatment in secondary care. Several GPs mentioned that they took this responsibility seriously to select patients needing further examination and possible treatment in secondary care (Quotes B1 and B2). Thus, based on a societal perspective, some GPs discussed the difficulty of referring many patients with joint pain for assessment by a rheumatologist (Quotes B1 and B2).

Deciding when to refer depended on the participants’ knowledge about the given disease. One GP observed that, generally, the referral rate is higher to medical specialties in which they do not feel confident about their knowledge (Quote B3). No drivers were discussed in relation to this theme.

#### Theme (C) difficulties in the referral of patients to rheumatologists

All participants expressed knowledge of the treat to target approach for RA, and regarded it as important. Some participants found the collaboration unproblematic (Quotes C1 and C2), whereas others experienced problems in collaboration with hospital rheumatologists, as they needed to argue why they had referred a patient (Quote C3). In particular, when the clinical picture was not ‘clear-cut’ from the GP’s perspective, the collaboration was sometimes seen as a hurdle (Quote C4). Some experienced easier access when patients were rheumatoid factor (RF) or anticyclic citrullinated peptide (ACPA)-positive (Quotes C4 and C5). Some felt they were reproved by hospital rheumatologists when they referred patients, which made them refer patients to a private practice rheumatologist instead (Quote C6). Some GPs used private practice rheumatologists when they did not suspect an inflammatory rheumatological disease but rather fibromyalgia, for example, and they only referred to rheumatologists at a specialized unit when they suspected inflammatory rheumatic disease (Quote C7).

Another barrier occurred when a patient had been seen earlier by a rheumatologist without a rheumatological diagnosis being given. The GP then regarded the patient’s complaint as non-rheumatological and would not seek a second opinion from a rheumatologist again (Quote C8).

#### Theme (D) laboratory tests—can they be trusted?

There were different understandings and interpretations of the laboratory tests for arthritis.

Some trusted their clinical view more than biomarkers (Quote D1), while others placed more trust in biomarkers and used them to rule out the diagnosis (Quote D2). General opinions were that ACPA is a much better biomarker than RF and that ACPA has eased the diagnostic process (Quotes D3 and D4). The limitations of using ACPA were, however, also discussed, including the fact that only some patients with RA are or will become sero-positive. One participant requested more information about that (Quote D5).

## Discussion

### Principal findings

We found that the GPs commented that RA is a difficult diagnosis to make. The overarching theme was *Like finding a needle in a haystack.*

The GPs found several barriers to diagnosing RA. The initial challenge is that RA symptoms often resemble more common and less severe conditions. Further barriers are maintaining the GP’s gatekeeper function, suboptimal collaboration with rheumatologists, and limitations in laboratory tests. The latter was especially challenging when the clinical picture was not ‘clear-cut’ from the GP’s perspective. If the patient had been seen by a rheumatologist before without being given an inflammatory rheumatic diagnosis, it could also be a barrier for re-referral.

The GPs suggested education, more specific tests, and good collaboration with and easy access to rheumatology departments as possible facilitators for diagnosing RA. They also recommended some future drivers, such as demonstration videos on how to perform clinical examinations for arthritis.

### Strengths and weaknesses of the study

A strength of focus group interviews is to gather in-depth information from different participants on a specific topic. This study has, however, some limitations. First, we only included GPs in the Central Denmark Region. However, as we had a broad range of participants in both age and experience, we had a heterogeneous group, thus giving the study a high external validity. However, we cannot exclude, the possibility that the GPs who chose not to participate in the study would have had other views on the subject. Potentially, they might have been more skeptical toward collaboration with rheumatologists, which would have resulted in higher perceived barriers in our study. Nevertheless, the included GPs were reimbursed for participation, and saturation was met after the last focus group interview in the sense that no new topic emerged [21]. A fourth focus group interview might have been beneficial, as it could have acted as a validation of the other three interviews. However, data saturation is about depth of data rather than about the numbers of interviews [21]. Also, data saturation is depended on sampling and how well a researcher manages to include participants that are able to contribute with relevant information related to the aim of the study. Most likely data saturation was attained relatively fast in our study because prompt diagnosis is a prevailing subject, which has a general appeal to many GPs. We thus believe that the group was representative enough to draw conclusions, even though we acknowledge that a fourth focus group interview might have been beneficial, as it could have acted as a validation of the other three interviews.

Second, the disadvantages of a heterogeneous group are the possibility of power imbalances [22]. However, the moderator’s style was non-directive, open-ended questions were used [22], and the moderator strived to let everyone be heard so that views on the subject were voiced by less dominant participants. Hence, we do not believe this had any significant impact on our results. Closed questions were only used, when there was a need for clarification of statements.

Third, there was evidently a difference in the moderating role from the first to the third focus group interviews, as the moderator became more experienced. Nevertheless, as the type of questions and the number of participants in the discussion were the same during the three interviews, we believe this had little influence on the results of the interviews. Furthermore, the researchers’ backgrounds can affect both the focus group interview and its analysis [19]. However, the professional background and clinical experience differed between the moderator and the observer. This gave different perspectives, predefined values, and theories, which we believe contributed positively to the study’s quality. Additionally, we used an inductive approach, which is a process of coding the data without trying to fit it into a preexisting coding frame or the researchers’ analytic preconceptions [19]. The study may have benefitted from a more experienced GP as an observer. However, experts from both primary care (MBJ) and rheumatology (EMH) were included in the study group, and they both played an active role in the interpretation of the results. Thus, we expect the impact of this to be insignificant.

### Findings in relation to other studies

To the best of our knowledge, these facilitators for the referral process have not been addressed in other studies, although they must be considered important factors to facilitate the diagnostic process for patients suspected for RA in the future.

We primarily used two qualitative studies from Flanders and Portugal for comparison [12,23]. The Flanders study was a qualitative study on the general practice perspective on early RA management, including 13 Flemish GPs [12]. The study from Portugal included 34 key stakeholders in a RA framework to address the patient’s access to RA healthcare and treatment. Of the thirty-four persons interviewed, ten were GPs [23].

We found that the GPs looked at arthritis and RA as challenging diagnoses, which corresponds with existing knowledge. Another qualitative study found that GPs had difficulties with suspecting RA in patients, as their symptoms in the early disease phase may be vague and difficult to distance from more common and non-inflammatory complaints [12,23]. GPs tended to think of early RA only when a patient already showed advanced joint inflammation [12]. That opinion also existed in our interviews, where the GPs found it difficult to diagnose when the patients did not present with classical signs ‘like in the textbook.’ This is in line with a cross-sectional survey of 1388 English GPs, which found that patient history had the greatest impact on the decision to refer, and, further, that clinical features, such as small joint swelling and pain, were most influential in diagnosing RA [24].

The GPs in our study saw the gatekeeper function as very important. This was not discussed in the studies from Flanders and Portugal [12,23], possibly due to the different healthcare systems’ organization. In Belgium, all specialists are accessible without referral [25]. In Portugal, however, patients need a GP referral to access other medical specialties, except for emergencies and out-of-hours visits [26]. Notably, the GPs we interviewed described barriers to early diagnosis that may conflict with the gatekeeper role, i.e. if they refer a much greater number of patients for suspected RA. Thus, gatekeeping can have an important drawback, as it may hamper timely diagnosis and treatment of patients suffering from a rare disease, especially if patients present with common symptoms [27].

An earlier Norwegian qualitative study has described that GPs often perceive referral to specialist care as an asymmetric process based on little dialog between specialist care and primary care[28]. This was not specifically mentioned by the GPs in our study, however some articulated that referral to rheumatology specialists was perceived as more difficult when the clinical manifestation was not clear-cut from the GPs’ perspectives. The same was seen in a Portuguese study, which found that the waiting time for a specialist was decreased if there was a strong suspicion of RA [23]. In our study, some GPs said that the referral process depended on the collaboration experienced in earlier cases. The Flemish study also highlighted that the choice of center for referral was influenced by the expected level of collaboration from that center’s rheumatology team [12].

The GPs in our study observed that the introduction of ACPA had had a positive impact on the diagnostic process. There are many citations regarding the laboratory tests as drivers. In the Portuguese study, laboratory tests were also discussed; the GPs believed that the availability of ACPA would improve the referral of patients with suspected RA to a specialist [19]. However, it can be a problem to rely too much on the ACPA result, as a patient can have negative RA serology despite a RA diagnosis [29]. In total, of those who will develop RA, 50–65% of patients are positive, and the positive predictive value of these tests in the general population is low, as the prevalence is low [29]. This was discussed in our interviews, but in general the barriers did not dominate the discussion regarding laboratory tests, although it is important to know the limitations thereof.

#### Meaning of the study

The present study provides a better understanding on facilitators of and barriers to the diagnostic interval in patients with RA in a Danish setting. Patients with early arthritis are a very heterogeneous group, and the initial symptoms often resemble those of more common and less serious conditions. This issue of vague/unspecific symptoms is also well described for early referral of cancer patients [30]. The final diagnosis is made by rheumatologist [1], thus fast and smooth cooperation across sectors will improve the prognosis for the patients, and therefore a close corporation and partnership between GPs and specialist care is essential. Future cross-sectoral research should focus on these facilitators and barriers, and on tools that can support first-line diagnosis in general practice, easy access to specialist advice, education, decision aid tools and point of care ultrasound.
